# Assessment of Lifestyle Self-Efficacy and Knowledge of Myocardial Infarction Symptoms Among Hypertensive Adults in Saudi Arabia: A Cross-Sectional Study

**DOI:** 10.3390/healthcare14183049

**Published:** 2026-09-17

**Authors:** Fahad Beshr Almughirah, Aseel Abdulhafitd Alharbi, Khalid Mohammed Aloufi, Mahmood Khaled Musa, Tariq Jameel Alrehaili, Mujtaba Hussain Almadani, Turky Ataallah Alsuhaimi, Mohammed Bandar Mujlid, Hussein M. Ismail, Muayad Albadrani

**Affiliations:** 1 College of Medicine, Taibah University, Madinah 42353, Saudi Arabia; 2 Department of Medicine, College of Medicine, Taibah University, Madinah 42353, Saudi Arabia; 3 Department of Family and Community Medicine and Medical Education, College of Medicine, Taibah University, Madinah 42353, Saudi Arabia; 4 Health and Life Research Center, Taibah University, Madinah 42353, Saudi Arabia

**Keywords:** hypertension, myocardial infarction, symptom awareness, self-efficacy, lifestyle modification, self-care, cardiovascular health literacy, Saudi Arabia

## Abstract

**Background/Objectives:** Hypertension is a major modifiable risk factor for cardiovascular disease worldwide and greatly contributes to myocardial infarction (MI), which is among the most severe and life-threatening forms of coronary artery disease. Despite progress in treatments, effective hypertension management depends on continuous lifestyle modifications and vigilant monitoring of acute heart symptoms. This study aimed to evaluate how confident patients are in implementing lifestyle changes and their knowledge of MI symptoms among those with hypertension in Saudi Arabia. **Methods:** A cross-sectional study was conducted from February to December 2025 involving adult hypertensive patients recruited from community and outpatient settings across Saudi Arabia. Data were collected using convenience sampling and a pretested questionnaire that included the Self-Efficacy subscale from the Hypertension Self-Care Profile, along with a 12-item scale measuring knowledge of MI symptoms. The associations among variables were analyzed using nonparametric tests and multivariable linear regression, with *p*-values under 0.05 considered statistically significant. **Results:** A total of 523 individuals with hypertension participated in the study. Their average lifestyle self-efficacy score was 50.33 ± 12.24, indicating moderate confidence in implementing lifestyle changes. The average score for knowledge about MI symptoms was 3.47 ± 3.84 out of 12, showing limited awareness. Being female and having comorbidities were significantly associated with higher knowledge levels (*p* < 0.05). Conversely, older age was significantly related to higher confidence levels (*p* < 0.05). Regression analysis indicated that both the confidence and knowledge models had limited explanatory power. **Conclusions:** The findings show that hypertensive patients have moderate confidence in managing their condition but lack sufficient awareness of MI symptoms. This highlights the need for targeted educational programs that promote long-term lifestyle adjustments and improve recognition of urgent cardiac symptoms to reduce cardiovascular illness and death.

## 1. Introduction

Cardiovascular disease encompasses coronary heart disease, cerebrovascular disease, peripheral artery disease, and aortic atherosclerosis. Hypertension and heart failure are among the most prevalent forms of CVD, with hypertension acting as a primary modifiable risk factor for MI. Coronary artery disease (CAD) is mainly caused by decreased myocardial perfusion that causes angina due to ischemia and can result in myocardial infarction (MI) in addition to heart failure. The World Health Organization (WHO) estimates that about 20 million people die each year from cardiovascular disease (CVD), making up nearly 32% of all global deaths [1,2]. Among the various modifiable risk factors contributing to cardiovascular disease, hypertension is a key cardiovascular risk factor that significantly accelerates vascular damage and raises the chances of MI and stroke [3,4].

In Saudi Arabia, hypertension affects nearly 30% of adults, and a significant proportion of cases remain undiagnosed or uncontrolled, reflecting current challenges in disease management [5,6]. Uncontrolled hypertension often coexists with other cardiometabolic risk factors such as obesity, smoking, and dyslipidemia, contributing to greater CVD risk and complicating blood pressure control efforts [7]. The problem of acute coronary syndromes (ACS) in Saudi Arabia further highlights this challenge; national assessments indicate that a significant cause of death is due to ACS, with treatment delays still common. Up to 40% of patients do not reach recommended reperfusion time targets, and in-hospital death remains significant.

Beyond clinical outcomes, effective hypertension management also depends on patients’ circumstances regarding healthy behaviors. Although clinical guidelines highlight lifestyle modification, including dietary change, physical activity, and smoking cessation, adherence to self-care recommendations is often low. Self-care behaviors and self-efficacy are closely linked, and low self-efficacy has been associated with poorer self-care adherence and higher complication risk among hypertensive patients [8,9].

Ischemic heart disease, which includes MI, with more than 9 million annual deaths, contributes significantly to this burden [2]. MI occurs when coronary blood flow is acutely interrupted, typically following atheromatous plaque rupture with subsequent thrombus formation, leading to myocardial ischemia and irreversible tissue injury in the absence of timely reperfusion.

Similarly, a good understanding of MI symptoms is essential to reducing pre-hospital delays and improving survival rates. Although chest pain is well known as a key symptom, awareness of other standard and unusual signs varies [2,10,11]. Awareness of atypical and less prominent symptoms remains incomplete across populations. Recent studies highlight a research and knowledge gap in this area, highlighting the need for strategic educational interventions, particularly for high-risk groups [8,12]. Hypertensive individuals constitute a particularly vulnerable subgroup given their elevated baseline cardiovascular risk, yet data examining their level of symptom awareness remain limited [13,14]. Previous studies in Saudi Arabia have documented hypertension prevalence and general cardiovascular knowledge separately, but few have evaluated both lifestyle self-efficacy and myocardial infarction symptom knowledge within the same high-risk hypertensive population [15]. Integrating these dimensions is necessary for developing comprehensive prevention strategies that address both long-term behavioral risk modification and rapid event responsiveness. Although the importance of self-efficacy for lifestyle changes and symptom awareness in acute cardiovascular events is well established, research in Saudi Arabia has rarely examined these areas collectively. More evidence is needed among hypertensive patients to inform prevention strategies that incorporate both long-term risk management and immediate response behaviors [16].

Consequently, this study aims to assess patients’ confidence in executing recommended lifestyle modifications and to evaluate their knowledge and awareness of MI symptoms among hypertensive individuals in Saudi Arabia.

## 2. Subjects and Methods

### 2.1. Study Design and Setting

A cross-sectional study was conducted in Saudi Arabia between February and December 2025. The study targeted community-dwelling adults diagnosed with primary hypertension across the Kingdom, recruited from community and outpatient settings, including chronic disease outpatient clinics. The objective was to assess patient confidence in implementing lifestyle changes and their knowledge of MI (heart attack) symptoms following hypertension diagnosis.

### 2.2. Study Population and Sampling

Eligible participants were male and female adults aged 18 years or older with a confirmed diagnosis of primary hypertension, recruited as a community and outpatient convenience sample across Saudi Arabia. Eight trained data collectors, coordinated by Taibah University, Madinah, Saudi Arabia, administered the electronic questionnaire to eligible patients between February and December 2025. Because enrollment records captured the response timestamp and the data-collector identifier rather than the recruiting facility, participation could not be attributed to individual hospitals or clinics. According to a national analysis of Ministry of Health Population Health Observatory records, approximately 1.72 million adults in Saudi Arabia had documented hypertension in 2025, corresponding to about 6.5% of the adult population [17]; this registered caseload is substantially lower than survey-based national prevalence estimates [5], indicating that a large share of affected adults remain undiagnosed or outside regular follow-up care. Of the 641 patients approached, 118 were excluded: 43 refused participation, 31 had incomplete questionnaires, 27 had secondary hypertension, 9 had language barriers, and 8 had incomplete demographic data. The final sample of 523 patients therefore represents a diverse community and outpatient cross-section of the broader hypertensive population in Saudi Arabia.

#### 2.2.1. Inclusion Criteria

- Adults aged 18 years or older residing in Saudi Arabia.
- English or Arabic speakers.
- Confirmed diagnosis of primary hypertension.
- Willing to participate in the study.

#### 2.2.2. Exclusion Criteria

- Secondary hypertension due to renal disease, primary hyperaldosteronism, pheochromocytoma, Cushing’s syndrome, hyperthyroidism, or other documented secondary causes.
- Incomplete questionnaire responses.
- Refusal to participate.
- Language barrier preventing comprehension of the questionnaire.

### 2.3. Sample Size Calculation

The sample size was calculated using a standard formula for cross-sectional studies, assuming a 95% confidence level, a 5% margin of error, a significance level of 0.05, and a population proportion (*p*) of 50% to maximize variability. The sample size was calculated using the following formula:

*n* = [*DEFF* × *Np*(1 − *p*)]/[(*d*^2^/*Z*21 − *α*/2(*N* − 1)+ *p*(1 − *p*)]

where: n = sample size and Z = 1.96 standard deviation for 95% CI. *p* = 50% and d = 5%. The minimum required sample size was 385 participants. A total of 523 hypertensive patients were included in the final analysis.

### 2.4. Data Collection and Study Instruments

Data were collected using a structured questionnaire divided into three sections: sociodemographic and clinical characteristics, confidence in lifestyle modification, and knowledge of heart attack symptoms.

No leading questions were included in the questionnaire. All items were neutrally worded to minimize response bias. Also, alcohol consumption was not assessed, as it is prohibited in Saudi Arabia and culturally sensitive; reported rates would likely be unreliable.

#### 2.4.1. Confidence in Lifestyle Modification

Confidence in managing hypertension was assessed using the validated Self-Efficacy subscale of the Hypertension Self-Care Profile [18]. This 20-item tool evaluates confidence in behaviors such as physical activity, dietary modifications, reducing salt and fat intake, medication adherence, blood pressure monitoring, weight management, quitting smoking, stress management, and scheduling regular doctor visits. Each item was evaluated using a 4-point Likert scale (1 = not confident, 4 = extremely confident). Total scores varied from 20 to 80, with higher scores reflecting greater self-efficacy. While the validated scale assesses broad lifestyle domains, specific behaviors, such as meal timing, were not included because the tool was designed to measure confidence in general self-care behaviors rather than detailed dietary patterns.

#### 2.4.2. Knowledge of Heart Attack Symptoms

Knowledge and awareness of MI symptoms were evaluated with a 12-item self-developed questionnaire adapted from prior studies. This questionnaire covered both typical and atypical symptoms, including chest pain, radiating pain, dyspnea, sweating, dizziness, nausea, fatigue, indigestion, palpitations, and anxiety. Responses were categorized as “Yes,” “No,” or “Not sure.” Correct answers received a score of 1, while incorrect or “Not sure” responses scored 0. The total score could range from 0 to 12.

### 2.5. Pilot Testing and Reliability

Both questionnaires were translated into Arabic using forward–backward translation to ensure linguistic accuracy and conceptual consistency. The 12-item knowledge questionnaire was developed systematically. Items were drawn from a comprehensive literature review of validated MI symptom awareness scales, including instruments from the WHO MONICA project, prior regional studies, and the American Heart Association’s published symptom lists. An initial pool of 15 items was reviewed by a panel of three cardiologists, two family medicine consultants, and one cardiovascular nurse educator at Taibah University to establish content validity. Experts rated each item on a 4-point scale (1 = not relevant to 4 = highly relevant) for relevance, clarity, and comprehensiveness. Items with an Item-Content Validity Index (I-CVI) ≥ 0.78 were retained, yielding the final 12-item version. Face validity was confirmed during pilot testing with 32 hypertensive patients, who reported the items as clear and understandable. Formal construct validation through factor analysis was not feasible given the sample size; however, the items correspond to established theoretical domains of myocardial infarction symptom recognition, distinguishing between typical symptoms (e.g., chest pain, radiating pain) and atypical symptoms (e.g., indigestion, fatigue). The translated version of the 20-item Self-Efficacy subscale of the Hypertension Self-Care Profile demonstrated good reliability (Cronbach’s α = 0.846), with item-total correlations ranging from 0.025 to 0.742. The 12-item knowledge scale demonstrated acceptable reliability (Cronbach’s α = 0.725), with item-total correlations ranging from −0.016 to 0.595. The item on “indigestion/epigastric discomfort” showed a negative correlation (−0.016); however, the overall scale remained within the acceptable range for internal consistency. The final analysis excluded pilot study data.

### 2.6. Reliability and Item Analysis

The internal consistency of the 12-item knowledge questionnaire was assessed using Cronbach’s alpha. Item-total correlations were examined to assess the contribution of each item to the overall scale. For any item showing a negative item-total correlation, Cronbach’s alpha was recalculated after excluding that item to evaluate its effect on the overall reliability of the questionnaire. The decision to retain or remove an item was based on its statistical contribution to scale reliability as well as its clinical relevance and contribution to the content coverage of myocardial infarction symptom knowledge.

### 2.7. Data and Statistical Analysis

Data were entered into Microsoft Excel (2016) and subsequently analyzed using IBM SPSS Statistics version 26. Descriptive statistics were computed for all variables, with categorical data presented as frequencies and percentages, and continuous data presented as mean ± standard deviation (SD) or median with interquartile range (IQR). We assessed normality using the Shapiro–Wilk test. The Mann–Whitney U test was used to compare two independent groups (e.g., gender, smoking status). In contrast, the Kruskal–Wallis test was used to compare three or more groups (e.g., age categories, marital status). *p*-value < 0.05 was considered statistically significant. Multivariable linear regression analyses were conducted to identify independent predictors of confidence in lifestyle modification and knowledge of heart attack symptoms. Prior to conducting multivariable linear regression analyses, we assessed key model assumptions. Multicollinearity was evaluated using Variance Inflation Factor (VIF), with VIF values < 2.0 indicating no significant collinearity among predictors. Linearity was assessed by visual inspection of partial regression plots and confirmed by the Durbin–Watson statistic (values close to 2.0, indicating independent errors). Homoscedasticity was evaluated using scatter plots of standardized residuals versus standardized predicted values. Normality of residuals was assessed using Q-Q plots and confirmed by the Shapiro–Wilk test (*p* > 0.05). Influential observations were examined using Cook’s distance; all values were <1.0, indicating no influential cases requiring exclusion. All assumptions were met, confirming the appropriateness of linear regression analysis.

As this was a survey-based cross-sectional study, participants were not required to answer all questions. Most variables had complete data for all 523 participants, including demographic characteristics (gender, nationality, education, employment, insurance, marital status, children, comorbidities, and smoking status), as well as all confidence and knowledge questionnaire items. However, age was missing for three participants (n = 520), as three respondents did not report their exact age. Blood pressure measurements were available for only 397 participants (75.9% of the sample), as these were self-reported and were not consistently documented for all participants. Given the limited extent and random nature of missing data, pairwise deletion was used for non-parametric analyses (Mann–Whitney U and Kruskal–Wallis tests), and listwise deletion was applied in multivariable linear regression models. This approach is consistent with standard practices for handling missing data in survey-based cross-sectional studies when missing data are minimal and missing at random [19].

Unstandardized regression coefficients (B), standardized regression coefficients (B), 95% confidence intervals (CI), and *p*-values were reported. The coefficient of determination (R^2^) was used to assess model fit.

### 2.8. Ethical Considerations

This study was approved by the Research Ethics Committee at the College of Medicine, Taibah University, under approval number [STU-25-021]. All participants were informed of the study’s purpose, their voluntary participation, and their right to withdraw at any time without consequences. No identifiable personal information was collected, and all responses were kept strictly confidential and used solely for research purposes. Data were securely stored and accessed only by the research team.

## 3. Results

### 3.1. Baseline Characteristics

A total of 641 hypertensive patients were approached for participation; 523 were included in the final analysis (response rate: 81.6%). The mean age of participants was 45.14 ± 15.36 years (range: 18–90 years), with a median of 47 years. The largest age group was 18–39 years (32.1%), followed by those aged 50–59 years (25.4%), 40–49 years (23.3%), and ≥60 years (19.2%). The sample was relatively balanced by gender, with 52.6% males and 47.4% females. The vast majority were Saudi nationals (96.2%). Nearly half of the participants were employed (46.8%), while 31.9% were unemployed and 21.2% retired. Governmental health insurance covered 75% of the sample, with the remaining 25% having private insurance. Most participants were married (65.8%), followed by single (23.9%), divorced (6.1%), and widowed (4.2%). Regarding family size, 34.6% had 3–5 children, 27.9% had no children, 27.5% had more than five children, and 9.9% had 1–2 children. More than half of the participants (54.7%) had no comorbidities, while 21.8% had one comorbidity, 13.8% had two, 6.5% had three, and 3.3% had four or more comorbid conditions. Blood pressure measurements were available for 397 participants, among whom the majority had uncontrolled hypertension: 39.5% were classified as Stage 2 hypertension, 34.3% as Stage 1 hypertension. In contrast, 10.8% had elevated blood pressure, and only 15.4% had normal blood pressure. Finally, the majority of participants were non-smokers (76.9%), with 23.1% reporting current smoking. All details are presented in Table 1.

### 3.2. Confidence in Lifestyle Modification and Knowledge of Heart Attack Symptoms

The mean confidence score among hypertensive patients was 50.33 ± 12.24 (median of 49 (IQR 18) out of 80), indicating a moderate level of confidence overall. The mean knowledge score was low at 3.47 ± 3.84 (median of 2 (IQR 6) out of 12), indicating fewer than one-third of the recognized heart attack symptoms (Table 2).

### 3.3. Factors Associated with Confidence in Lifestyle Modification

Significant associations were observed with confidence level and age, gender, and smoking status in Table 3. Confidence scores increased progressively with age (*p* = 0.004), with median scores rising from 45 (16) among those aged 18–39 years to 50 (19) among those aged 40–59 years, and 51.5 (18) among participants aged 60 years and above. Females demonstrated significantly higher confidence, with a median of 51 (18), compared to males (*p* = 0.021). Additionally, non-smokers reported significantly greater confidence, with a median of 50 (18) than smokers (*p* = 0.005).

### 3.4. Factors Associated with Knowledge of Heart Attack Symptoms

Age was strongly associated with knowledge (*p* = 0.002), with median knowledge scores increasing from 1 (4) among the youngest group to 3.5 (7) among those aged ≥60 years. Females also demonstrated significantly better knowledge, with a median of 3 (7), than males (*p* = 0.005). Marital status showed a strong association (*p* < 0.001), with widowed (median 5 (4)) and divorced (median 5.5 (8)) participants demonstrating the highest knowledge. The number of children was also significant (*p* = 0.002), with knowledge increasing from those with no children (median 1 (4)) to those with >5 children (median 4 (7)). Finally, the presence of comorbidities was strongly associated with better knowledge (*p* < 0.001).

Table 4 presents the multivariable linear regression analysis examining factors associated with confidence in lifestyle modification among hypertensive patients. The model explained 7.1% of the variance in confidence scores (R^2^ = 0.071). No included variable reached statistical significance at the conventional *p* < 0.05 threshold. Age demonstrated a borderline association with confidence (B = 0.123, β = 0.158, *p* = 0.051), indicating a trend toward higher confidence among older patients. Employment status approached significance (B = 2.544, β = 0.102, *p* = 0.061), suggesting employed individuals may report greater confidence compared to unemployed or retired participants. Knowledge of heart attack symptoms showed a trend toward association with confidence (B = 0.309, β = −0.084, *p* = 0.056). The standardized coefficients for these variables ranged from −0.084 to 0.158, indicating small effect sizes. The remaining variables, including gender, nationality, insurance status, marital status, number of children, comorbidities, blood pressure, and smoking, did not demonstrate significant associations with confidence scores (*p* > 0.05).

Table 5 displays the multivariable linear regression analysis examining factors associated with knowledge of heart attack symptoms. The model accounted for 6.6% of the variance in knowledge scores (R^2^ = 0.066). Female gender was significantly associated with higher knowledge scores (B = 0.920, β = 0.120, *p* = 0.008). Employment status demonstrated a significant negative association (B = −0.826, β = −0.056, *p* = 0.023), indicating that unemployed and retired participants had lower knowledge compared to employed individuals. The presence of comorbidities was significantly associated with better knowledge (B = 0.937, β = 0.105, *p* = 0.019). Marital status showed a borderline association (B = 0.403, β = 0.012, *p* = 0.076). Age, nationality, insurance status, and number of children did not reach statistical significance (*p* > 0.05). The standardized coefficients ranged from −0.056 to 0.120, reflecting small to modest effect sizes for the significant predictors. Collectively, these findings suggest that while demographic and clinical factors contribute to knowledge about MI symptoms, they explain a limited proportion of the variance in awareness.

## 4. Discussion

This study shows that hypertensive patients in Saudi Arabia demonstrate moderate confidence in managing their condition through lifestyle modifications but have insufficient awareness of MI symptoms.

Cardiovascular diseases are increasingly impacting more people worldwide, with their incidence rising each day. These are complex conditions that can appear suddenly or develop gradually. A thorough understanding of these diseases among patients is essential for providing optimal care. Most existing research on lifestyle changes has concentrated on individual behaviors, such as quitting smoking [20]. However, progress in lifestyle modification varies across the main modifiable risk factors, including dyslipidemia, obesity, physical inactivity, hypertension, and diabetes [21].

Using a validated, structured questionnaire, the research examined both behavioral self-efficacy in managing hypertension and awareness of emergency cardiac signs among hypertensive adults recruited from community and outpatient settings. We evaluated Saudis’ ability to recognize heart attack signs and to enhance their knowledge of MI and hypertension, aiming to reduce related morbidity and mortality. Prior research has identified a substantial lack of CVD knowledge among the Saudi public, including studies from regions such as the South, West, and Jeddah City consistently [22,23,24,25].

Our results showed moderate confidence in lifestyle management, with an average self-efficacy score of 50.33 ± 12.24. This suggests that although patients feel capable of making changes such as improving their diet, sticking to medication routines, and increasing physical activity, their confidence may not be sufficient for sustained behavioral change. Similar findings have been observed in other hypertension cohorts. For instance, a study in Indonesia using the Hypertension Self-Care Profile reported moderate self-care practices, with a mean score of 49.78 ± 6.64. This indicates that while many patients understand lifestyle recommendations, maintaining long-term adherence remains difficult [26]. However, if people are not sufficiently aware of acute cardiac symptoms, confidence in lifestyle management alone may not prevent serious cardiovascular events. Therefore, assessing patients’ awareness of MI warning symptoms is essential, especially among those with hypertension who have an increased risk of coronary events. From our results, awareness of MI symptoms was insufficient, achieving a mean score of 3.47 ± 3.84 out of 12, signifying that participants recognized fewer than one-third of the symptoms.

These findings are consistent with previous research demonstrating gaps in awareness of MI warning signs across many populations. A global systematic review including 124 studies from 35 countries reported that although chest pain is the most widely recognized symptom of MI, awareness of other symptoms, such as jaw pain, back pain, or light-headedness, remains considerably lower worldwide [27].

Similarly, a population-based study in Korea found that only 42.4% of participants were aware of the main symptoms of MI. Recognition of atypical signs, such as arm, shoulder, jaw, or neck pain, was particularly low. In Saudi Arabia, studies showed relatively high awareness of typical symptoms such as chest pain (87.1%) and shortness of breath (86%), but awareness of less common symptoms, such as indigestion, dizziness, and fatigue, was significantly lower [8].

Our findings are consistent with global patterns of limited MI symptom awareness. A systematic review of 124 studies across 35 countries reported that although chest pain is widely recognized, awareness of other symptoms (jaw pain, back pain, light-headedness) remains considerably lower [27]. Population-based studies from Korea, Iran, and Malaysia similarly documented recognition rates of typical symptoms ranging from 70–90% but significantly lower recognition of atypical presentations [28,29,30]. This international pattern suggests that inadequate identification of non-standard cardiac symptoms is a global challenge, potentially leading to delayed emergency interventions and increased likelihood of unfavorable outcomes. The particularly low awareness levels observed in our high-risk hypertensive cohort warrant urgent attention.

We also observed a significant link between age and confidence in lifestyle management. However, the multivariable regression model in this study explained only 7.1% of the variance in confidence scores and 6.6% for knowledge, indicating that demographic and clinical factors alone do not adequately explain variability in self-efficacy or symptom awareness among hypertensive patients. These R^2^ values reflect the limited predictive capacity of our measured sociodemographic variables. The remaining unexplained variance may be attributable to factors not captured in our study. Based on previous research, we propose that educational attainment and health literacy levels, duration of hypertension diagnosis, personal or family history of cardiovascular events, quality of physician-patient communication and counseling received, medication adherence patterns, socioeconomic status, and social support networks may substantially contribute to the remaining variance. Studies have demonstrated that self-efficacy is shaped by complex interactions between patient characteristics and healthcare system factors, including health literacy and social support [31], and that these psychosocial determinants often explain more variance in self-care behaviors than demographic characteristics alone [32]. Future studies should incorporate comprehensive psychosocial measures, including validated health literacy assessments, social support scales, and detailed cardiovascular risk perception instruments, to better understand the determinants of self-efficacy and symptom awareness in this high-risk population.

Age, employment status, and the link between knowledge and confidence had *p*-values between 0.051 and 0.061 in our regression models. Those values are a bit above the conventional cutoff for significance. We are not treating these as solid associations. They do point to possible patterns, though. Larger studies with better psychosocial measures are needed to check if these trends are real.

Conversely, a comprehensive understanding of all MI symptoms was not achieved. These shared characteristics suggest that inadequate identification of non-standard cardiac symptoms persists as a global and regional concern, potentially leading to delayed emergency interventions and an increased likelihood of unfavorable outcomes. Attitudinal data derived from this investigation reveal that although a substantial number of participants expressed a willingness to pursue medical attention upon experiencing chest pain, a considerable proportion (46.5%) would postpone seeking assistance unless they were certain of an MI. Furthermore, approximately 70% of the respondents reported feeling embarrassed about presenting to a hospital without an apparent medical necessity [33].

These patterns indicate a complex link between recognizing symptoms and seeking medical help. Similar trends appear in population research. A prior survey in England showed that 51% of people lacked confidence in identifying common heart attack symptoms, with nearly half of the population feeling the same. Additionally, 36% of respondents indicated they would not call an ambulance if they or someone they knew experienced chest pain [34].

Our study did not collect data on shift work status, which is known to influence cardiovascular risk and potentially impact lifestyle self-efficacy. Future research should examine whether shift workers with hypertension differ in their confidence or symptom awareness.

These findings suggest that health awareness, particularly for individuals with diverse cardiac conditions and a risk of MI, should be guided by this data. Although awareness of classic symptoms remains high, many people still hesitate or hold misconceptions about emergency care, leading to delays in treatment and poorer outcomes. These insights into attitudes underscore the importance of not only enhancing symptom knowledge but also public health messages that shape behavioral intentions and emergency response attitudes.

### Practical Implications for Clinical Practice and Health Systems

Our findings suggest several practical approaches to enhance cardiovascular care for hypertensive patients in Saudi Arabia. At the clinic level, integrating structured education about heart attack symptoms and lifestyle confidence during routine visits can be effective. Simple tools like symptom checklists, teach-back methods, and brief educational sessions can reinforce knowledge. Hypertension specialist nurses are well-positioned to deliver this education through individual sessions, telephone follow-ups, and group programs, using motivational interviewing to address barriers to lifestyle change. Primary care should embed symptom recognition into annual hypertension reviews and use waiting room materials in Arabic. Electronic health records could flag patients with low knowledge for extra support.

Beyond clinical settings, community-based programs can extend reach through collaborations with the Ministry of Health, social media campaigns, and culturally tailored materials. Engaging religious leaders and community pharmacies could further disseminate information. At the health system level, embedding knowledge assessment into quality metrics and establishing referral pathways to health educators would support sustainability. Interventions should prioritize younger patients, males, smokers, and those without comorbidities, who showed lower knowledge or confidence. Pilot testing, cultural adaptation, and health literacy-sensitive materials are essential for effective implementation. These strategies, applied systematically, could reduce the cardiovascular disease burden in Saudi Arabia.

## 5. Limitations

This study has several limitations. First, the cross-sectional design precludes causal inferences about the relationships observed. Second, the community and outpatient convenience sampling approach may limit generalizability, and because enrollment records captured the data-collector identifier and response timestamp rather than the recruiting facility, facility-level enrollment counts could not be reported; the sample should therefore be interpreted as a community and outpatient convenience sample rather than a formally multicenter one. Third, self-reported data are subject to recall and social desirability biases. Fourth, the low R^2^ values in regression models indicate that unmeasured factors, such as social support, health literacy, or prior cardiovascular education, likely contribute to confidence and knowledge. Fifth, our study did not collect detailed data on specific lifestyle factors such as meal timing, late-night eating, or breakfast skipping, which may influence cardiometabolic risk and blood pressure control. Future research should incorporate comprehensive lifestyle assessments to capture these potentially overlooked behaviors, particularly given emerging evidence linking chrono-nutrition to hypertension and cardiovascular risk. Sixth, the knowledge scale, while demonstrating acceptable reliability, showed one item with a negative correlation. This may reflect poor recognition of atypical symptoms, item ambiguity, or translation issues. For instance, the Arabic term for ‘indigestion’ may be conflated with ‘heartburn’ or general abdominal discomfort, potentially affecting item interpretation. This issue warrants further psychometric testing across diverse populations, including formal factor analysis and differential item functioning assessment. Finally, the pilot sample used to assess questionnaire reliability was relatively small, and further psychometric testing across diverse populations is warranted.

## 6. Conclusions

Hypertensive individuals in Saudi Arabia had modest confidence in adopting lifestyle changes but possessed low awareness of MI symptoms. These findings highlight a significant disparity between confidence in chronic illness self-management and the detection of acute cardiovascular symptoms in a high-risk population. Incorporating structured cardiovascular education into hypertension management, emphasizing persistent lifestyle changes and early identification of MI symptoms, could enhance prompt healthcare-seeking behavior and decrease unnecessary morbidity and mortality. Additional longitudinal studies are needed to examine the psychosocial factors that affect self-efficacy and symptom awareness.

## Figures and Tables

**Table 1:** Sociodemographic and Clinical Characteristics of Saudi Hypertensive Patients [n = 523].

Factor	n (%) or Mean ± SD/Median (IQR)
Age (years), Mean ± SD [n = 520]	45.14 ± 15.36
Age(years), Median (IQR), Range	47 (24), 18–90
Age(years), n (%)	
18–39	167 (32.1)
40–49	121 (23.3)
50–59	132 (25.4)
≥60	100 (19.2)
Gender, n (%)	
Male	275 (52.6)
Female	248 (47.4)
Nationality, n (%)	
Saudi	503 (96.2)
Non-Saudi	20 (3.8)
Employment status, n (%)	
Employed	245 (46.8)
Unemployed	167 (31.9)
Retired	111 (21.2)
Insurance status, n (%)	
Governmental	392 (75)
Private	131 (25)
Marital status, n (%)	
Married	344 (65.8)
Single	125 (23.9)
Divorced	32 (6.1)
Widow	22 (4.2)
Number of children, n (%)	
No children	146 (27.9)
1–2	52 (9.9)
3–5	181 (34.6)
>5	144 (27.5)
Number of comorbidities, n (%)	
None	286 (54.7)
1	114 (21.8)
2	72 (13.8)
3	34 (6.5)
≥4	17 (3.3)
Blood Pressure, n (%) [n = 397]	
Normal	61 (15.4)
Elevated	43 (10.8)
Stage 1 HTN	136 (34.3)
Stage 2 HTN	157 (39.5)
Smoking, n (%)	
Yes	121 (23.1)
No	402 (76.9)

Valid percent is used for all percentages. Sample size (n) next to each factor is the number of reported data (e.g., Age n = 520, BP n = 397).

**Table 2:** Saudi Hypertensive Patient Confidence in Hypertension Lifestyle Modifications and Heart Attack Symptom Knowledge.

Factor	Mean ± SD	Median (IQR)	Range	Out of
Confidence Score	50.33 ± 12.24	49 (18)	21–80	80
Knowledge Score	3.47 ± 3.84	2 (6)	0–12	12

**Table 3:** Impact of demographics and baseline characteristics on patient confidence in hypertension lifestyle modifications and heart attack symptom knowledge.

Factor	Confidence Median (IQR)	*p*-Value	Knowledge Median (IQR)	*p*-Value
Age (years) [n = 520]		**0.004**		**0.002**
18–39	45 (16)		1 (4)	
40–59	50 (19)		2 (7)	
≥60	51.5 (18)		3.5 (7)	
Gender		**0.021**		**0.005**
Male	47 (18)		1 (6)	
Female	51 (18)		3 (7)	
Nationality		0.205		0.333
Saudi	49 (19)		2 (6)	
Non-Saudi	45.5 (11)		3 (8)	
Employment status		0.124		0.925
Employed	48 (19)		2 (7)	
Unemployed/Retired	49 (18)		2 (6)	
Insurance status		0.134		0.999
Governmental	49 (19)		2 (6)	
Private	48 (16)		2 (6)	
Marital status		0.657		**<0.001**
Married	49 (18)		2 (6)	
Single	46 (18)		1 (4)	
Divorced	49.5 (20)		5.5 (8)	
Widow	55.5 (23)		5 (4)	
Number of children		0.284		**0.002**
No children	46 (19)		1 (4)	
1–5	49 (18)		2 (6)	
>5	50 (18)		4 (7)	
Comorbidities		0.148		**<0.001**
Yes	50 (18)		4 (6)	
No	48 (19)		1 (5)	
Blood Pressure [n = 397]		0.450		0.532
Controlled/Elevated	49.5 (20)		1.5 (6)	
Uncontrolled	49 (19)		2 (6)	
Smoking		**0.005**		0.962
Yes	46 (16)		2 (6)	
No	50 (18)		2 (6)	

Bold *p*-values indicate statistical significance (*p* < 0.05).

**Table 4:** Linear regression of patient confidence in hypertension lifestyle modifications.

Factor	Unstandardized Coefficients B	Standardized β	95% CI	*p*-Value
Age (years)	0.123	0.158	0.000–0.246	0.051
Gender	1.668	0.068	−1.007–4.342	0.221
Nationality	−4.174	−0.061	−11.309–2.961	0.251
Educational Level	2.441	0.102	−0.237–5.119	0.074
Employment Status	2.544	0.104	−0.116–5.204	0.061
Insurance status	−1.273	−0.046	−4.122–1.577	0.380
Marital status	−0.438	−0.029	−2.013–1.137	0.585
Children	−1.062	−0.066	−3.565–1.441	0.405
Comorbidities	0.616	0.025	−2.267–3.499	0.675
Blood Pressure	−1.172	−0.042	−3.917–1.573	0.402
Smoking	−2.341	−0.084	−5.365–0.684	0.129
Knowledge Score	0.309	0.097	−0.008–0.626	0.056

**Table 5:** Linear Regression of Patient Knowledge of Heart Attack Symptoms.

Factor	Unstandardized Coefficients B	Standardized β	95% CI	*p*-Value
Age (years)	0.004	0.015	−0.030–0.038	0.824
Gender	0.920	0.120	0.245–1.595	**0.008**
Nationality	0.207	0.010	−1.558–1.971	0.818
Educational Level	−0.448	−0.056	−1.194–0.298	0.239
Employment Status	−0.826	−0.107	−1.537–−0.114	**0.023**
Insurance status	0.108	0.012	−0.672–0.888	0.786
Marital status	0.403	0.083	−0.042–0.849	0.076
Children	0.544	0.105	−0.132–1.220	0.115
Comorbidities	0.937	0.122	0.156–1.719	**0.019**

Bold *p*-values indicate statistical significance (*p* < 0.05).

## Data Availability

The raw data supporting the conclusions of this article will be made available by the authors on request.
